# Protocol for a hybrid II study exploring the feasibility of delivering, evaluating, and implementing a self-management programme for people with neuromuscular diseases at a specialist neuromuscular centre

**DOI:** 10.1186/s40814-022-01231-9

**Published:** 2023-01-09

**Authors:** Laurence Edward Lee, Stefan Tino Kulnik, Geoffrey M. Curran, Annette Boaz, Gita M. Ramdharry

**Affiliations:** 1 Department of Neuromuscular Diseases, UCL Queen Square Institute of Neurology, London, UK; 2 Ludwig Boltzmann Institute for Digital Health and Prevention, Salzburg, Austria; 3 Departments of Pharmacy Practice and Psychiatry, University of Arkansas for Medical Sciences, Little Rock, USA; 4 Faculty of Public Health and Policy, The London School of Hygiene & Tropical Medicine, University of London, London, UK

**Keywords:** Implementation science, Self-management, Neuromuscular diseases, Normalisation process theory, Co-design, Stakeholder engagement

## Abstract

**Background:**

Self-management support (SMS) forms a central pillar in the management of long-term conditions. It is firmly aligned with UK health policy but there is a paucity of evidence exploring how it is enacted in the context of neuromuscular diseases (NMDs). Bridges is a SMS programme originally developed in stroke. A new version of the programme (Neuromuscular Bridges) has recently been co-designed with people with lived experience of NMD and requires evaluation.

The implementation of SMS is inherently complex with potential barriers at the level of the patient, provider, and wider organisation. The success of implementing programmes can be highly dependent on context, indicating a rationale for considering implementation determinants at an early stage. This study aims to explore the feasibility of (1) delivering, (2) evaluating, and (3) implementing Neuromuscular Bridges at a specialist neuromuscular centre.

**Methods:**

This study employs a hybrid II design underpinned by Normalisation Process Theory (NPT), which has been used prospectively to inform the implementation plan and will also inform the analysis. The feasibility of delivering, evaluating, and implementing Neuromuscular Bridges will be assessed using a single-arm pre-post design. In terms of delivery and evaluation, we will explore acceptability, demand within the service, performance of outcome measures, recruitment, and retention.

Implementation strategies have been selected from a refined taxonomy of strategies, mapped to NPT, and targeted at known barriers and facilitators at the specialist centre that were identified from preliminary stakeholder engagement activities. The impact of the strategy bundle on fidelity, acceptability, appropriateness, and adoption will be evaluated using qualitative interviews, administrative data, surveys, and a notes audit.

**Conclusions:**

This this study will provide valuable feasibility data on a co-designed SMS programme for people with NMDs that will be used to inform a larger implementation study, requirements for embedding it in a specialist centre, and rollout to other specialist centres. Using hybrid methodology at the feasibility stage is unusual and this study will provide important insights into the usefulness of taking this approach at this point in the research pipeline.

**Trial registration:**

ISRCTN Trial ID: ISRCTN14208138. Date registered: 18/08/2021.

## Introduction

Neuromuscular disease (NMD) is a broad term encompassing a wide range of conditions that impact on the functioning of muscles. Many NMDs are lifelong conditions, and common symptoms include progressive muscle weakness, degeneration and wasting, and sensory impairment. Individually, many NMDs are considered rare diseases, affecting less than 5 in 100,000 people [1]. However, collectively the prevalence of NMD is significant, affecting approximately 70,000 people in the UK [2].

Living with NMD can also result in significant psychological burden. Depressive symptoms [3], reduced social participation [4, 5], perceived loss of control [6], and negative effects on relationships [7] have all been reported. Fatigue is a common presenting symptom [8, 9], and people with NMDs can experience distress from the challenges of obtaining a diagnosis, and the uncertainty surrounding disease progression [6]. The multi-system nature of these disorders, and their need for complex long-term multidisciplinary care, acts as a driver to developing long-term management strategies for individuals with these conditions [10].

Self-management support (SMS) is a chronic care model which forms a central pillar in the management of many long-term conditions and is firmly aligned with UK health policy [11]. It has been frequently associated with statistically and clinically significant improvements in conditions such as diabetes and hypertension [12], and there is a growing body of evidence to support its use in neurological conditions [13–17]. Despite this, SMS has had very little exploration in people living with NMDs. The constantly changing, complex, and unpredictable nature of NMDs is likely to result in a different set of self-management priorities for these individuals, as they try to adapt to an uncertain future.

The rarity of many NMDs, combined with their progressive, lifelong nature, means that the setup of the clinical services that support them are very different to other neurological settings. Neuromuscular care is provided through a hub and spoke model via specialist services. Within these specialist services patients are seen annually or bi-annually by a range of clinicians, including specialist clinical neurologists, neurophysiologists, neuropathologists, neurophysiotherapists, and specialist nurses. This care model is different to single-onset conditions such as stroke or traumatic brain injury, which have a relatively stable disease trajectory, and where patients have an intensive period of clinical input before being discharged. Conversely, people with NMD will usually remain under the care of a specialist centre indefinitely, due to the often progressive nature of neuromuscular conditions, and subsequent mounting care needs over the lifespan. This provides a clear rationale for developing our understandings of how SMS is enacted in this population and understanding the nuances of the context in which it is delivered.

Bridges is an individualised, evidence-based SMS programme that has been developed for people living with long-term conditions such as stroke and traumatic brain injury [14, 18]. In January 2020, a specially adapted version of Bridges specific to people living with NMDs was launched, Neuromuscular Bridges (NM Bridges) [19]. For the first time, the Bridges programme will contain a co-designed digital support application alongside its usual co-designed patient workbook. The setup of neuromuscular care in the UK is a new service structure for Bridges. The feasibility of NM Bridges within this service structure requires exploration, as will the acceptability and utility of the new digital format.

Understanding implementation determinants of the intervention should be considered as early as possible, and preferably in the trial design stage [20]. By attending to implementation processes, likely to be relevant to transitioning the intervention to practice settings, potential issues can be anticipated at the level of the patient, clinician, and the organisation delivering the intervention, with an appreciation for the political and financial environment, as well as the wider societal context [21]. This is especially important when considering SMS, which involves pathways of care with interdisciplinary input, behaviour change from multiple stakeholders, and can be highly influenced by environmental factors, as well as social determinants such as ethnicity or socio-economic status [22].

Hybrid trials, first proposed by Curran et al. [23], aim to simultaneously change practice and establish intervention feasibility/efficacy/effect, by taking a dual focus of testing both clinical and implementation outcomes. There is a growing body of evidence to suggest that hybrid designs speed the translation of knowledge into practice [24], and they have been employed in a wide range of different healthcare settings and patient populations, including breast cancer [25], mental health [26, 27], chronic pain [28], dementia [29], and critical care [30]. This mixed methods study will employ a “hybrid II approach”. The dual focus afforded by this design will allow us to test the feasibility of delivering and evaluating a new SMS intervention, NM Bridges, whilst simultaneously testing the feasibility of a combination of implementation strategies, from here on in referred to as an “implementation strategy package”.

## Objectives

1. To explore the feasibility of delivering and evaluating NM Bridges through a single, specialist service:
  - Feasibility of delivery of the NM Bridges intervention
    Explore acceptability of NM Bridges (to clinicians and participants) when delivered in one-off interactions (measured through qualitative interviews and analysis of patient reported outcome measures).
    Explore demand for NM Bridges within the service (explored through semi-structured interviews, uptake/engagement of clinicians with the intervention).
  - Feasibility of evaluation
    Examine the acceptability and performance of candidate outcome measures (measured through semi-structured interviews, analysis of the collected quantitative data).
    Explore participant recruitment and retention (explored through administrative data and assessment of completeness/quality of collected data).
2. To evaluate the *feasibility and preliminary impact of an implementation strategy “package”* to implement NM Bridges at a specialist neuromuscular centre:
  Monitor fidelity of the implementation process (*measured through educational team meetings*).
  Monitor intervention fidelity (*measured through session observation, a fidelity checklist, and educational meetings*).
  Explore acceptability of the implementation strategy package to clinicians at the specialist centre (*explored through semi-structured interviews, uptake/engagement of clinicians with the intervention, implementation outcome instruments*).
  Identify barriers and facilitators to the implementation strategy package (*explored through semi-structured interviews, implementation outcome instruments*).
  Explore appropriateness and practicability of the implementation strategy package to the specialist clinical service (*explored through semi-structured interviews, uptake/engagement of clinicians with the intervention, implementation outcome instruments*).
  Monitor effect of implementation strategy package on adoption (*measured through semi-structured interviews, administrative data*).

## Methods

### Participants and setting

The study will take place at Queen Square Centre for Neuromuscular Diseases, which is a research and clinical centre that specifically focuses on genetic and acquired NMDs. The service at Queen Square employs a team of specialist health professionals, including consultant neurologists, therapists, and nurses, who have expertise in diagnosing and managing muscle and nerve conditions. They offer a comprehensive range of specialist multidisciplinary services and clinics. The centre provides clinical services for over 5000 patients per year and is based in the National Hospital for Neurology, Queen Square, UCLH Foundation NHS Trust.

Participants will be eligible if they are as follows: (i) age > 18 years; (ii) currently a patient at Queen Square Centre for Neuromuscular Diseases or UCLH; (iii) have a diagnosis of neuromuscular disease from a neurologist at the Queen Square Centre for Neuromuscular Diseases; (iv) have the capacity to give informed consent to participate in the research. This study has been registered with the ISRCTN registry (trial ID: ISRCTN14208138).

### Study design

We will be using an embedded experimental mixed methods design, to address the research questions, as this is an often used design when evaluating health services research [31]. An embedded design, where one data set provides a supportive secondary role to the other, is frequently used when researchers need to include a qualitative component within a quantitative study. The use of qualitative methods post-intervention will enable us to follow up on the participants’ experiences of the intervention, and the experiences of staff delivering the intervention [32]. This approach will enable us to gain an understanding of how the intervention fits in with the day to day workings of the clinical centre, the perceived value it provides to patients, and provide data on potential barriers and facilitators to the uptake of the intervention [33].

The feasibility of the intervention will be tested through a single-arm pre-post design, with participants completing outcome measures before, directly after, and 3 months post-intervention. A randomised control trial, which is a higher design in the traditional hierarchy of evidence, was considered for this work. However, it was felt that randomisation at the patient level could be problematic due to the potential for intervention contamination when clinicians are transitioning back and forth between providing (a) routine care or (b) the intervention, for different patients.

It is now widely accepted that addressing the challenge of implementing evidence-based interventions into routine practice is of fundamental importance to the global health community, and implementation science is regarded as a vital tool in closing the evidence-to-practice gap [20]. The consideration of implementation components in research is not new. For example, Bowen et al. [34] recommend employing feasibility studies to study general areas such as acceptability, adaptation, and demand, which clearly have overlap with established implementation outcomes. However, a consideration for implementation components in feasibility studies is not always reflected in the literature. In this study, we propose using a hybrid methodology to permit a dual focus on both feasibility testing of the intervention, and feasibility testing of the implementation strategy package. It is hypothesised that this design, will allow us to interrogate the implementation aspects of this trial in a more structured, coherent, and rigorous way, whilst simultaneously gathering valuable data on the clinical intervention. At the end of the study, we will reflect on the value of using this approach at the feasibility stage for future implementation studies.

Feasibility data from this study will be used to inform future implementation efforts in the form of a cluster randomised study, as ultimately implementation questions would benefit from more sites to include different systems and infrastructure, to address potential limits on generalisability. Prior to setting this up, there will be scoping work required at potential sites to understand the nuances of delivery that may differ. This will allow us to collaboratively design the trial with the partner sites to explore the implementation and ensure fidelity within the different contexts. This exploratory study will provide information for us to refine delivery of the intervention at “treating” sites, for comparison with control sites where usual treatment will be provided. We are aware that there are limitations of the pre-post design and that missing information would need to be substituted/estimated from other available studies; or estimated from data collected later on, for example during an internal pilot phase of a definitive cluster RCT.

### Theoretical underpinning

This study design has been informed by Normalisation Process Theory (NPT) [35], a socio-behavioural theory focused on the “social organisation of the work (implementation), of making practices routine elements of everyday life (embedding) and of sustaining embedded practices in their social contexts (integration)” [35]. It is frequently used in implementation research and explores factors influencing sustainable changes in practice. As implementation of SMS is inherently complex and represents a move away from usual ways of working, NPT will provide a framework through which to assess facilitators and barriers to the incorporation of NM Bridges into routine practice, to the point of becoming “normalised” within the setting [36]. In this study, NPT will be used to (1) provide focus on the everyday work of clinicians to provide explanation to how the intervention is embedded, (2) give insight into how the intervention fits with the procedures involved in usual care, and (3) explain variations in implementation processes (not just focussing on barriers and facilitators). NPT has been employed in previous studies exploring the implementation of self-management support and complex health-care interventions, even at the feasibility stage [14, 36–40]. In this study, it has been invoked prospectively to inform the study design, it will be used in the analysis to identify where the intervention is addressing potential issues in implementation, and it has been used to develop materials such as the topic guides for qualitative interviews exploring implementation factors.

### Stakeholder engagement

Patient and public involvement (PPI), and wider stakeholder engagement, has been a central focus of this body of work since its inception, ensuring that the research is focussed on issues that are relevant and meaningful to people with NMD, and ensuring they have a say in how the research is designed, undertaken, and disseminated. At the beginning of the project, a PPI group was formed which included 8 people with NMD from the neuromuscular clinics at the specialist centre. The group have been involved in several design aspects of the study, including the content of the topic guides, the selection of outcome measures, and the wording and content of the participant documentation. The PPI group has supported the applications for ethical approval and continue to be a valuable resource for understanding the patient perspective for this body of research.

In order to design an implementation strategy package that was contextually sensitive, it was important to obtain data related to the experiences and preferences of people working in the target setting [41]. As such, preliminary work with key stakeholders in the team was carried out at an early stage in the trial design. This involved three meetings with the core team of six clinicians, and a mix of nursing staff and allied health professionals. By actively engaging clinicians at the specialist centre at this stage, consulting them on what the potential barriers to implementing NM Bridges might be, and the strategies that were required to address them, it was hoped that the relevance, transparency, and usefulness of the implementation plan could be enhanced. Crucially, these factors have been linked with both positive implementation and clinical outcomes [42–44]. As the clinical team were the intended knowledge-users, research suggests that this “front-end” gathering of opinions may result in the selection of implementation strategies that are more appropriate and feasible in the intended system and organisational context [41, 43]. Finally, by establishing a culture of collaboration and involvement with the clinical team early on, it was hoped that a sense of “shared ownership” would be created, subsequently enhancing engagement with the evaluation of NM Bridges.

This stakeholder engagement work played a significant part in the development of the study protocol. The study implementation strategies were selected based on the barriers and facilitators described by clinicians at the centre, as were decisions surrounding preferences for communication, and the recruitment process.

### NM Bridges intervention

The NM Bridges intervention is an individualised, person-centred self-management approach which is delivered to patients by trained clinicians within clinical interactions as part of routine practice [45]. It aims to support the integration of self-management support into clinical appointments through professional interactions and treatments. The approach is theoretically underpinned by Social Cognitive Theory [46], which describes the influence of individual experiences, the actions of others, and environmental factors on individual health behaviours. Self-efficacy, which is routinely associated with improved self-management outcomes [47], forms a critical construct within the approach. However, Bridges is not just concerned with concepts such as personal agency and learning theories. As the approach has evolved, it has increased its emphasis on understanding how organisational processes such as standardised goal setting methods and outcome measures may act as barriers to supporting self-management [48]. This focus on the effect of structural issues is coherent with emerging understandings that emphasise the context-dependent nature of self-management programmes, and the notion that the delivery of complex interventions, i.e. healthcare interventions that include several active separate critical components, is further complicated when they are situated within complex settings [49–51].

Fundamental to the Bridges approach is a bespoke, interdisciplinary training workshop for healthcare professionals. The workshops are tailored according to the clinical setting and clinical caseload and focus on reshaping communication styles, using language modifications and facilitation techniques that can help to promote self-management in a non-didactic way. The training provides clinicians with strategies to help patients to build capability and confidence to pursue quality of life goals and emphasises the importance of creating an environment which is inclusive, open, and collaborative [52]. During the Bridges training, clinicians are equipped to support patient self-management using seven key principles (Table 1).

**Table 1:** Seven key principles of the Bridges self-management programme

Reflection: Attributing changes and progress to personal effort, not the skills of the healthcare professional Self-discovery: Finding new ways of doing things and trying out different activities and strategies. Goal setting: Avoiding clinician-led goals, focussing on patient priorities and what is meaningful and relevant. Encouraging small steps to promote feelings of success and working towards longer-term aspirational goals. Accessing resources: Using available resources to achieve personal goals. Including their own past skills and experiences Problem solving: To come up with different ideas, strategies, and ways to adjust, rather than relying on suggestions from healthcare professional Activity: Encouraging any activity, however small. Knowledge: Knowledge about their condition, but also about what works for their own situation and challenges.	

Co-creation and co-design constitute key principles in the development and delivery of successful self-management interventions, and Bridges has longstanding expertise in working to these principles [53]. A central pillar of the approach is the emphasis placed on the co-design of context-specific peer support tools, such as the NM Bridges patient workbooks. For the new NM Bridges intervention, a series of co-deign workshops, interviews, and focus groups were conducted with patients with NMDs, their families, and healthcare professionals from the specialist neuromuscular service. This led to the development of a co-designed workbook and digital app to support self-management, which the participants named “Adapting to life with a neuromuscular condition”. These resources contained patient stories, exercise tips, advice for family and friends, clinicians answering frequently asked questions, useful resources, and space to reflect and record progress.

The process of intervention tailoring to the organisational and population context also included co-designing the training workshop for clinicians. Two sessions were held involving people with lived experience of NMD and a multidisciplinary sample of staff from the specialist centre. The co-design process explored the priorities of these stakeholders and explored context-specific considerations for NM Bridges that were subsequently woven into the training package. For example, strategies for maximising the potential of the intervention within a one-off session, as in previous studies the Bridges intervention was delivered as part of a series of rehabilitation sessions [14, 54, 55]. Practically, in this study NM Bridges will be delivered to patients as part of routine therapy and nursing appointments at the specialist centre, with the co-designed resources reinforcing the intervention content.

### Implementation strategy procedures

An implementation strategy package will be employed concurrently with the intervention as part of the hybrid II design. This has been informed by the ImpRes framework [56], which provides a systematic step-by-step approach to designing implementation research. Using this framework has ensured that the rationale for the selection and tailoring of the implementation strategies was both robust and appropriate. The framework guided tailoring of the study-specific educational material, and the process for systematic identification of implementation determinants. The choice of strategies was informed by known barriers and facilitators, identified through pre-trial consultation with stakeholders within the specialist service. The strategies were selected from the Cochrane EPOC Taxonomy of Implementation Strategies [57] and are established elsewhere in the literature [58–60]. This selection and tailoring to a specific context has key face validity [20], and a Cochrane review found that strategies tailored to contextual needs were more effective in improving professional practice than no intervention or dissemination of guidelines [61].

Implementation champions will be identified through the training process to support staff who are less familiar with self-management interventions. They will have one to one meetings with the lead researcher as required, to discuss any challenges and identify where the lead researcher may need to provide additional support to staff delivering the intervention. It is not anticipated that this will require significant additional time, but they will be well placed “on the ground” to be detect if any other colleagues are struggling, through usual daily interactions of the team.

Further description of the implementation strategies, along with their theoretical basis, the proposed measurement techniques, and mapping to known barriers and facilitators to implementation identified in earlier stakeholder engagement work can be found in Tables 2 and 3. The tables also describe how strategies have been mapped to NPT. For example, how iterative development of educational material and the co-design of the educational package for clinicians is anticipated to create a sense of “collective action”. Specification of individual implementation strategies is highly recommended by the Expert Recommendations for Implementing Change (ERIC) framework [59, 62], and specification of the individual strategies can be found in Table 4.

**Table 2:** Barriers identified in preliminary stakeholder engagement activities

Barriers	Imp. strategies	NPT construct	Imp. outcomes	Measurement
Lack of time limiting opportunities to support self-management	NM Bridges **tailored** to context. **Inter-professional education** to highlight benefits of working in this way. **Academic detailing** to describe evidence-base, and benefit to patients & the wider healthcare system.	**Coherence**: Illustrating the difference between NM Bridges and established staff interactions with patients. Education provided on the underpinning theory and illustrating its practical application. **Differentiation:** understanding the difference between NM Bridges, and more traditional approaches to patient communication.	Fidelity, acceptability, adoption rates, organisational feasibility.	Observation, checklists, self-report, semi-structured interviews, AIM, FIM, NoMAD
One-off interactions (as opposed to multiple rehab sessions)	Iterative development of **educational material in monthly cycles**, co-design of the educational package for clinicians, **continuous quality improvement** through ongoing remote support and education from the research team	**Collective action**: Consideration, discussion, and action planning for specific demands of the specialist neuromuscular service. Integration into current service processes through team meetings, goal setting, documentation. **Initiation:** The staff’s motivation in trying to incorporate NM Bridges into their clinical practice.	Fidelity, acceptability, technical feasibility	Semi-structured interviews, AIM, FIM, NoMAD
Increased time requirement to complete self-reported fidelity checklist	**Educational meetings and local consensus processes,** where fidelity is discussed alongside problem solving strategies and adaptions to mitigate evolving barriers	**Cognitive participation:** Accommodating professionals’ shared and differing beliefs. Collaborative methods for incorporation of NM Bridges into ways of working.	Fidelity, acceptability, technical and organisational feasibility	Self-report, semi-structured interviews, AIM, FIM, NoMAD
Potential for decreased implementation momentum due to caseload	**Audit and feedback** from patients using NM Bridges to be provided to staff via weekly email to promote engagement. **Reminders:** Bi-monthly emails sent from the research team to remind staff about NM Bridges and to encourage further adoption.	**Collective action:** Facilitating engagement through flexible training slots, availability of material such as patient workbooks, staff peer mentoring. **Reflexive monitoring (reconfiguration):** Suggestions from participants that aim to modify and enhance the utility of the NM Bridges programme.	Fidelity, acceptability, adoption rates, organisational feasibility, appropriateness	Observation, checklists, self-report, semi-structured interviews, AIM, FIM, IAM, NoMAD

**Table 3:** Facilitators identified in preliminary stakeholder engagement activities

Facilitators	Imp. strategies	NPT construct	Imp. outcomes	Measurement
Intervention matches well with the mission and philosophy of the service	**Educational meetings:** promoting cognitive participation by emphasising the benefits to patients, practices and healthcare system (coherence). Identify and prepare **NM Bridges “champions”** to actively and enthusiastically promoting the intervention.	**Coherence (internalised meaning**). The coherence of NM Bridges will be derived from the meaning users collectively invest in it. **Communal specification: t**he process through which clinicians, share and create an understanding of this new practice will be explored. **Cognitive participation (legitimation)**: The belief that NM Bridges is right for the context, in terms of being a needed adjunct to existing tools and approaches will drive engagement	Acceptability, organisational feasibility, appropriateness	Self-report, semi-structured interviews, IAM, AIM, NoMAD
The flexibility afforded by the NM Bridges intervention	**Educational meetings and educational materials** to reinforce the flexibility of the intervention and its underlying collaborative philosophy, ability to be tailored within consultations	**Reflexive monitoring (communal appraisal):** regarding the outcomes and values of NM Bridges. **Collective action (relational integration):** to enhance participant’s understandings of NM Bridges. Not only how and when to use the Bridges approach, but also understanding the expressions and concerns of other staff members	Fidelity, acceptability, adoption rates	Observation, checklists, self-report, semi-structured interviews, AIM, FIM, IAM, NoMAD
The perceived effectiveness & usefulness of NM Bridges by the service	Identify and prepare **NM Bridges “champions”** to actively promote the intervention, mobilise resources, communicate how the intervention fits into a vision for the larger organisation. **Educational meetings and educational materials** to reinforce mechanisms that the intervention targets and provide opportunity for dialogical reflection on processes.	**Reflexive monitoring (systematisation):** The participants’ judgement of NM Bridges regarding usefulness and effectiveness will be monitored and used to drive implementation and engagement. Communal appraisal. **Communal and individual appraisal** regarding the outcomes and values of NM Bridges.	Fidelity, acceptability, technical feasibility, adoption, appropriateness	Observation, checklists, self-report, semi-structured interviews, AIM, FIM, IAM, NoMAD

**Table 4:** Specification of implementation strategies

	**Strategy 1: Clinician implementation team meetings**
Actor(s)	The team of clinicians who are implementing the NM Bridges intervention
Action(s)	Reflect on the implementation effort, share lessons learned, support learning, and propose changes to be implemented in small cycles of change.
Target(s) of the action	Clinicians newly trained in the intervention.
	Knowledge about how to use the intervention in this context, intentions to use the innovation, social influences.
Temporality	First meeting should be within two weeks of initial training.
Dose	Once monthly for 1 h for the duration of the trial.
Implementation outcome(s) affected	Uptake of the intervention, penetration among eligible clients/patients, fidelity to the protocol of the clinical innovation, sustainability of the innovation.
Justification	Cooperative learning theory [63]
	**Strategy 2: NM Bridges “champion” (develop stakeholder interrelationships)**
Actor(s)	Individual clinician in the clinical setting
Action(s)	Facilitating reflection; serving as team leader; motivating staff; engaging in planning activities; persuading staff that the initiative was important and worthwhile; building relationships with key stakeholders; using data to persuade peers
Target(s) of the action	Clinician newly trained in the intervention
Temporality	Ongoing input throughout trial, MDT meetings, flexibility to respond and tailor as appropriate
Dose	Ongoing input throughout trial
Implementation outcome(s) affected	Uptake of the innovation, fidelity to the protocol of the clinical innovation, acceptability to clinicians.
Justification	Advocacy for clinical champions with literature [57, 60, 64]
	**Strategy 3: Adapt and tailor to context**
Actor(s)	Intervention development team
Action(s)	Conduct co-design activities to tailor NM Bridges training to clinical setting and population
Target(s) of the action	Clinicians newly trained in the intervention
	Knowledge about how to use the intervention in this context, intentions to use the innovation, social influences.
Temporality	Co-design sessions to take place 1 month prior to delivery of training, allowing time for appropriate modification of training and materials
Dose	2 co-design sessions prior to staff training in intervention
Implementation outcome(s) affected	Uptake of the intervention, penetration among eligible clients/patients, acceptability to clinicians, fidelity to the protocol of the intervention, appropriateness of intervention to context
Justification	Research suggests that tailoring interventions to a particular context has positive effect on healthcare outcomes [61, 65]
	**Strategy 4: Educational materials**
Actor(s)	Local opinion leaders (key members of clinical team)
Action(s)	Email provision of NM Bridges resources, links to Bridges webinars, supporting literature, examples of successful use of intervention to include patient success stories
Target(s) of the action	Clinicians newly trained in the intervention
	Knowledge about how to use the intervention in this context, intentions to use the innovation
Temporality	First educational email within 2 weeks of start of trial
Dose	Weekly email to provide updates, encouragement, and feedback on intervention implementation
Implementation outcome(s) affected	Uptake of the intervention, acceptability to clinicians, fidelity to protocol
Justification	Theoretical Domains Framework (Reinforcement, environmental context & resources, social influences) [66]

#### Fidelity

Fidelity of NM Bridges will be assessed through ten session observations by the research team, a self-reported fidelity checklist to be completed by clinicians after each session, and educational meetings/discussions. Ten intervention sessions will be video recorded and reviewed by the research team, who will evaluate the session against pre-determined fidelity markers. Sessions for observation/recording will be selected at random. All session observations and recordings will only take place with the informed consent of both the patient and clinician.

### Outcomes

#### Feasibility of the clinical intervention

Outcome measures related to the intervention will be completed at baseline, directly after receiving the intervention and 3 months afterwards. There will be the option for outcome measures to be completed remotely, for participant convenience, and to reduce risk associated with the COVID-19 pandemic. The outcome measures will focus on the intervention’s impact on health-related quality of life (EQ-5D [67]), self-efficacy (GSES [68]), mood [General Health Questionnaire (GHQ [69])], and participation (Ox-PAQ [70]). A profile of the broad impacts of the intervention will be measured through the Health Education Impact Questionnaire (heiQ) [71], which covers areas that patients, clinicians, policymakers, and researchers regard as important outcomes of patient education programs for people with long-term conditions. The person-centredness of the intervention will be measured through the Client-Centred Care Questionnaire [72], and the and Patient Assessment of Chronic Illness Care (PACIC) [73].

It is recommended that patient engagement is considered in the selection of outcome measurements [74], and as such, the outcome measures were selected based upon discussion with the project’s PPI group, who emphasised the need to consider the biopsychosocial impact of NMD, not just the physical elements. Once the views of people with lived experience of NMD had been obtained, measures were selected based on literature providing recommendations on the use of outcome measures for self-management interventions [22, 75], rare neurological conditions [76], and person-centred care [77, 78].

#### Feasibility of implementation

As recommended by the ImpRes framework [56], and Proctor et al. [79], a clear mechanism for measuring the effect of the implementation strategies on pre-defined implementation outcomes has been established. A mixed methods approach will also be employed for this aspect of the study. Quantitative measures were chosen from the Implementation Outcome Repository, based on a systematic review by Khadjesari et al. [80]. These will include (a) the Acceptability of Intervention Measure (AIM) [81], (b) Intervention Appropriateness Measure (IAM) [81], and (c) Feasibility of Intervention Measure (FIM) [81] which are four-item measures of implementation outcomes that are often considered “leading indicators” of implementation success [79]. We will also use the NoMAD scale [82, 83] which is a pragmatic, psychometrically tested instrument that measures implementation activities related to NPT. Table 4 describes the theoretical basis and techniques that will be used to measure the impact of the main implementation strategies on implementation outcomes.

Qualitative methods will be employed concurrently to gain a deeper insight into the opinions, thoughts, and feelings on NM Bridges of participants. Ten patients will be recruited for semi-structured interviews to explore their experiences of receiving NM Bridges. Six staff will be interviewed at the end of the intervention phase to investigate determinants of implementation behaviour and explore experiences of delivering NM Bridges. Ten patients is a pragmatic number in terms of funding, staffing, and time frame as this study forms part of a PhD project. There will be six staff interviews as this is how many staff members have been trained in the approach and subsequently delivering the intervention. The topic guide for patients has been informed by previous qualitative work into patient perspectives on self-management [84]. The topic guide for clinicians has been designed by mapping questions to the four constructs of NPT. Interviews will be audio recorded and transcribed by an external service, and a reflexive thematic analysis undertaken [85].

### Planned sample size and analyses

A target sample of 60 people with NMD will be recruited through the clinical service and neurology clinics at the Centre for Neuromuscular Diseases. A sample size of at least 30 is recommended for early feasibility studies, but a larger target has been set to account for potential dropouts and for secondary exploration of the performance and suitability of the outcome measures. We will collect data on screening, recruitment, and drop out reasons for all participants. The recruitment rate will be calculated from the total number of participants recruited over the recruitment window. This will be expressed as numbers recruited per month. Descriptive statistics will be used to describe averages at baseline and following the intervention. Continuous data will be presented as a mean, with standard deviation or median and interquartile range, as appropriate. For categorical/ordinal data, we will calculate frequencies and percentages. The effect of the intervention will be explored by calculating the adjusted Cohen’s *d* effect size statistic for continuous outcome measures or the adjusted Hedge’s G for ordinal outcomes [86].

Qualitative data in the form of semi-structured interviews will be audio recorded and transcribed verbatim and a reflexive thematic analysis will be conducted. The transcripts will be entered into the qualitative software management programme NVivo V.12, to facilitate data management. The qualitative analysis will be conducted by one researcher (LL) and discussed/reflected in the wider group of researchers and with the PPI advisory group (peer review). There is also a plan for independent double-coding of data.

The principal analysis approach will be deductive, answering to the aims of interviewing, i.e. to explore the participants’ experiences of the intervention and the experiences of staff delivering the intervention, with the possibility for inductive development of any further codes/themes should these be identified. There will be an assessment in how far qualitative findings match the results from quantitative measures (intervention outcomes, implementation measures). Qualitative findings will directly inform design decisions of the intervention and implementation strategies for a follow-on study.

In terms of success criteria, the study findings will be assessed and judged in context, i.e. recruitment rate in relation to patient volume of the service, and intervention effect by comparison with other positive trials of self-management interventions in other clinical populations.

### Dissemination

We will use a range of approaches to disseminate our findings. The results and analysis of the study will be disseminated to service users through publication and on the Muscular Dystrophy UK (MDUK) website. MDUK is the main charity for people with muscle wasting conditions in the UK. The study findings will also be disseminated to the other major NMD charities to share with their members and followers. The research team will be disseminating the results of the study through videoblogging on social media, to make the findings more accessible to individuals who prefer to interact using these platforms. Academic dissemination will include publications and presentations to researchers and healthcare professionals, and there will be an emphasis on service-user collaboration and public engagement in dissemination strategies. We will also work with the team at the specialist centre to develop strategies for disseminating findings and meet with them to discuss the study’s implications and next steps.

## Data Availability

Not applicable.
